# Retraction: Flavor-cyber-agriculture: Optimization of plant metabolites in an open-source control environment through surrogate modeling

**DOI:** 10.1371/journal.pone.0259294

**Published:** 2021-10-25

**Authors:** The PLOS ONE Editors

After this article [1] was published, concerns were raised about the article’s methods and reporting. PLOS followed up in accordance with COPE guidance and PLOS policies; this process involved a post-publication assessment with input from a statistical review and multiple members of *PLOS ONE*’s Editorial Board.

The following concerns were noted and/or confirmed during the post-publication editorial assessment:

1. The experimental methods and statistical analysis methods and models were not reported in sufficient detail to enable replication efforts and to clarify how potential confounds were addressed. The authors addressed some of the methodological reporting issues by providing additional information in post-publication discussions with PLOS and referring to materials posted on GitHub, but they did not fully address the questions about the statistical analyses.
2. An Editorial Board member raised concerns about the selection of machine learning model used in the study and noted that the article did not adequately discuss query learning literature.
3. Consulted experts advised that the dataset is too limited to address the article’s objectives, and that given the size of the dataset, one would expect issues with overfitting data to non-linear models.
4. Concerns were raised about the number of independent replicates included in the experiments. The authors provided the following information about replication:
  There was one treatment replicate per climate condition. For chemical analysis, four biological replicates (four basil plants) were sampled for each treatment replicate, but these were pooled before chemical and GC-MS analysis. For Rounds 1 and 3, one chemical measurement was taken for each treatment (pooled sample). For the Round 2 GC-MS experiments, three technical replicates of each pooled sample preparation were analyzed.
  An Academic Editor advised that considering known limitations of GC-MS and especially since the data were used for modelling, this study did not include sufficient biological and technical replicates to yield reliable results. Consequently, the Academic Editor advised that the article’s conclusions are not adequately supported.

The authors disagreed with points 2 and 3, above.

Based on the expert input received, including the issues discussed above about reporting, limitations of the dataset, and insufficient replication, the *PLOS ONE* Editors concluded that there are concerns about the reliability of the article’s results and conclusions, and overall, the article did not meet the journal’s third and fourth publication criteria. (The third and fourth publication criteria require that experiments must have been conducted rigorously, with appropriate controls and replication; methods must be reported in sufficient detail to allow others to reproduce the study; and conclusions must be supported by the data.) Therefore, the *PLOS ONE* Editors retract this article. We regret that the issues with this article were not identified during the pre-publication peer review process.

AJJ, EM, TLS, RM, and CBH did not agree with retraction and stand by the article’s findings. JdlP did not respond or could not be reached.

Note: the computational modelling code used in this study was not provided with the published article but has since been deposited in the GitHub repository. The authors clarified that surrogate-optimization methods used the DEAP framework of Fortin et al. [2].
